# Is there an association between endometriosis and subsequent breast cancer? A retrospective cohort study from Germany

**DOI:** 10.1007/s10549-023-07211-8

**Published:** 2023-12-23

**Authors:** Niklas Gremke, Sebastian Griewing, Jacob Göhring, Anna Isselhard, Uwe Wagner, Karel Kostev, Matthias Kalder

**Affiliations:** 1 Department of Gynecology and Obstetrics, University Hospital Marburg, Philipps-University Marburg, Baldingerstraße, 35043 Marburg, Germany; 2 Institute of Molecular Oncology, Philipps-University Marburg, Hans-Meerwein-Straße 3, 35043 Marburg, Germany; 3 Epidemiology, IQVIA, Frankfurt, Germany

**Keywords:** Endometriosis, Subsequent breast cancer, Gynecological practices, Germany

## Abstract

**Purpose:**

Given the relatively high incidence of both endometriosis and breast cancer, investigating the potential connection between these gynecological diseases is of substantial clinical significance. However, there is no clear consensus in the literature on the extent to which the risk of breast cancer is increased in patients with endometriosis. Therefore, we conducted a large-scale observational study investigating the association between endometriosis and breast cancer risk.

**Methods:**

This study included women aged ≥ 18 years with an initial endometriosis diagnosis from one of 315 office-based gynecologists in Germany between January 2005 and December 2021. Non-endometriosis patients were matched 1:1 to patients with endometriosis based on age, index year, average yearly consultation frequency, and predefined co-diagnoses within 12 months before or on the index date, including obesity and benign breast disorders. The association between endometriosis and the 10-year incidence of breast cancer was studied using Kaplan–Meier curves and log-rank tests. Finally, a univariable Cox regression analysis was conducted to assess the association between endometriosis and breast cancer.

**Results:**

Over a follow-up period of up to 10 years, no significant difference was observed between the endometriosis (2.4%) and the matched non-endometriosis group (2.5%) with regard to breast cancer diagnoses. Furthermore, the regression analysis revealed no significant association between endometriosis and subsequent breast cancer.

**Conclusion:**

In summary, our comprehensive 10-year study involving a substantial sample of women indicates that endometriosis is not significantly associated with an increased risk of subsequent breast cancer.

## Introduction

Endometriosis affects approximately 5–10% of women of reproductive age and is characterized by the presence of endometrial tissue outside the uterus [1]. Endometriotic lesions are typically located in the pelvic region and are most commonly found in the sacrouterine ligament, ovaries, and the peritoneum of the pouch of Douglas and of the bladder [2]. Based on the histopathology and location, endometriosis can be divided into three subtypes: superficial peritoneal lesions, deep infiltrating endometriosis (DIE), and ovarian cysts (endometriomas) [3]. The main proposed hypotheses for the etiology of endometriosis are retrograde menstruation theory (transplantation of endometrial cell into the peritoneal cavity) and coelomic metaplasia theory (transformation of peritoneal mesothelium into the endometrium). There is broad evidence that endometriotic lesions are initiated and sustained through a complex interplay of molecular processes, including inflammatory response, immune dysregulation, proangiogenic factors, and endocrine regulation [4]. Although endometriosis is considered a benign gynecological disease, the underlying pathophysiologic mechanisms and growth patterns of deep infiltrating endometriosis (e.g., invasion of surrounding structures) resemble those of a malignant disease [5].

Sampson was the first to describe the link between endometriosis and ovarian cancer in 1925, and the increased risk of cancer in patients with endometriosis was confirmed in a number of studies over the following decades [6, 7]. In particular, Pearce et al. conducted a large pooled analysis of 13 case–control studies and found that endometriosis was associated with a significantly increased risk of clear cell and endometrioid ovarian cancer (OR of 3.05, 95% CI 2.43–3.84 and OR of 2.04, 95% CI 1.67–2.48, respectively) [7], findings that were also supported by a number of further large meta-analyses [8, 9]. Given that endometriosis can lead to systemic pro-oncogenic changes, such as chronic inflammation [10, 11] and an abnormal hormonal environment [12], additional studies have also explored a potential association between endometriosis and distant cancers, such as breast cancer [13]. Notably, endometriosis and breast cancer also share many risk factors such as prolonged estrogen exposure from early menarche to late menopause and nulliparity [14, 15]. However, there are conflicting results in the literature regarding the association between endometriosis and risk of subsequent breast cancer. For example, in a meta-analysis of cohort and case–control studies, Ye and colleagues showed that women with endometriosis had an increased risk of breast cancer (RR of 1.082, 95% CI 1.001–1.169) [16]. By contrast, the systematic literature review and meta-analysis published previously by Gandini et al. did not demonstrate any increased risk of breast cancer among women with endometriosis (SRR 1.04, 95% CI 0.99–1.09) [17]. However, many studies are limited by their inclusion of self-reports (no medically confirmed diagnosis of endometriosis), a short observation period, and a small number of adjustment factors to reduce confounding. In light of these constraints, there is a compelling need for further evidence to elucidate the potential association between endometriosis and breast cancer. Therefore, the aim of this retrospective cohort study, which included 30,484 women, was to analyze the risk of breast cancer in endometriosis patients followed in 315 gynecological practices in Germany. The goal was to generate more evidence regarding the extent to which there is an increased risk of breast cancer among endometriosis patients.

## Methods

### Database

This retrospective cohort study was based on data from the Disease Analyzer database (IQVIA). This database has already been used in several previous studies focusing on breast cancer [18–22] and contains anonymized data on diagnoses and prescriptions as well as basic medical and demographic data from computer systems used in participating office-based practices [23]. The database includes data from approximately 3000 office-based practices in Germany. The sampling method for the Disease Analyzer database uses statistics from the German Medical Association to determine the panel design based on specialist group, German federal state, community size category, and physician age. It has previously been shown that the panel of practices included in the Disease Analyzer database is representative of general and specialized practices in Germany [23].

### Study population

**Fig. 1:**
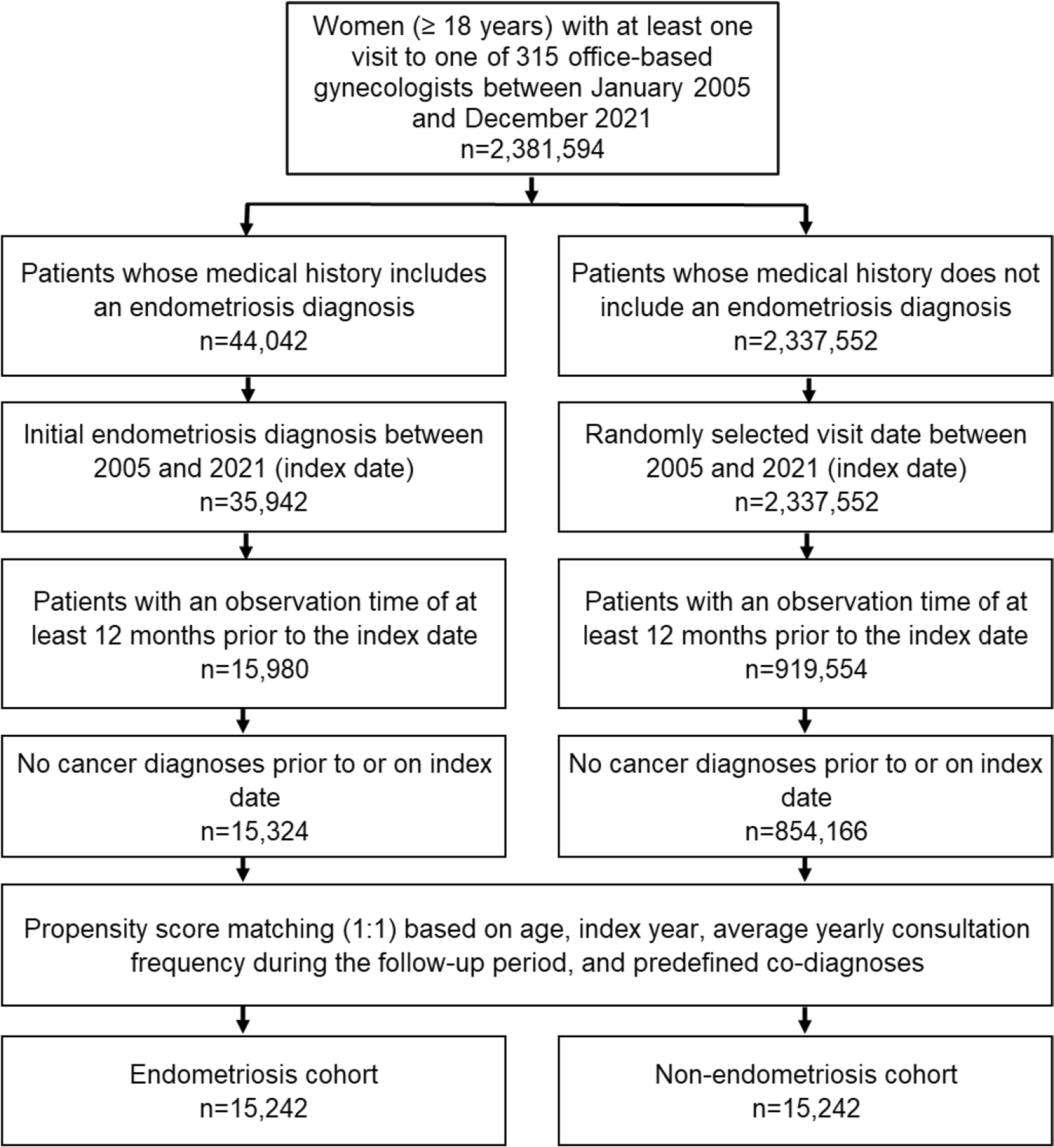
Selection of study patients

This study included women aged ≥ 18 years with an initial endometriosis diagnosis (ICD-10: N80) from one of 315 office-based gynecologists in Germany between January 2005 and December 2021 (index date; Fig. 1). One further inclusion criterion was an observation time of at least 12 months prior to the index date. Patients with cancer diagnoses prior to or on the index date were excluded. After applying similar inclusion criteria, women without endometriosis diagnoses were matched to women with endometriosis using greedy propensity score matching (1:1) based on age (± 1 year**)**, index year, average yearly consultation frequency during the follow-up, and predefined co-diagnoses documented within 12 months prior to or on the index date, obesity (ICD-10: E66), disorders of the breast including benign dysplasia, hypertrophy, inflammation, or unspecified lump (ICD-10: N60–N65), and benign, in situ or uncertain behavior neoplasms of the breast (ICD-10: D05, D24, D48.6, D49.3). For the non-endometriosis cohort, the index date was that of a randomly selected visit between January 2005 and December 2021 (Fig. 1).

### Study outcomes and statistical analyses

The outcome of the study was the initial diagnosis of breast cancer (ICD-10: C50) up to ten years following the index date as a function of endometriosis. Differences between the endometriosis and non-endometriosis cohorts in terms of sample characteristics and diagnosis prevalence were compared using the Wilcoxon signed-rank test for continuous variables, the McNemar test for categorical variables with two categories, and the Stuart-Maxwell test for categorical variables with more than two categories.

The 10-year cumulative incidence of breast cancer in the cohort with and without endometriosis was also examined using Kaplan–Meier curves, and these curves were compared using the log-rank test. Finally, a univariable Cox regression analysis was conducted to assess the association between endometriosis and breast cancer. The results of the Cox regression model are displayed as hazard ratios (HRs) and 95% confidence intervals (CIs). Cox regression analyses were also conducted separately for four age groups (≤ 30, 31–40, 41–50, > 50 years). These age groups were classified based on age distribution among endometriosis patients (90.3% are ≤ 50 years old). Due to the multiple comparisons and large patient samples involved in this study, a *p* value of < 0.01 was considered statistically significant. Analyses were carried out using SAS version 9.4 (SAS Institute, Cary, USA).

## Results

### Basic characteristics of the study sample

The present study included 15,242 women with endometriosis and 15,242 women without endometriosis. The basic characteristics of the study patients are displayed in Table 1. The mean age was 35.7 (standard deviation (SD): 9.6–9.8) years, and more than 90% of patients were aged 50 years or younger. Patients visited their gynecologists an average of 3.2 times per year during the follow-up period. Due to the matched pairs design, no significant differences were observable between both cohorts in terms of age, visit frequency, or co-morbidities (Table 1).

**Table 1:** Baseline characteristics of the study sample (after propensity score matching)

Variable	Proportion among women with endometriosis (%) *N* = 15,242	Proportion among women without endometriosis (%) *N* = 15,242	*p*-value
Age (mean, SD)	35.7 (9.8)	35.7 (9.6)	0.820
Age ≤ 30	3852 (25.3)	3923 (25.7)	0.064
Age 31–40	5736 (37.6)	5682 (37.3)	
Age 41–50	4358 (28.6)	4231 (27.8)	
Age > 50	1296 (8.5)	1406 (9.2)	
Number of physician visits per year during the follow-up (mean, SD)	3.2 (3.0)	3.2 (3.0)	1.000
Diagnoses documented within 12 months prior to or on index date			
Obesity	1030 (6.8)	1030 (6.8)	1.000
Breast disorders (benign dysplasia, hypertrophy, inflammation, or unspecified lump)	4426 (29.0)	4426 (29.0)	1.000
Benign, in situ or uncertain behavior neoplasms of the breast	684 (4.5)	684 (4.5)	1.000

Proportions of patients given in *N* and % unless otherwise indicated

*SD* standard deviation

### Association between endometriosis and subsequent breast cancer

**Fig. 2:**
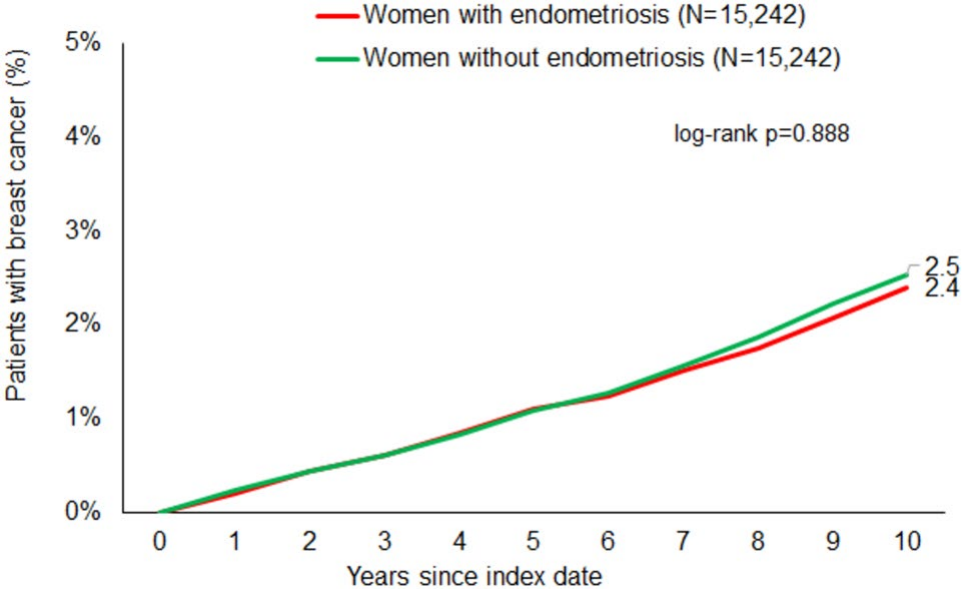
Cumulative incidence of breast cancer in individuals with and without endometriosis

After up to 10 years of follow-up, 2.4% of endometriosis patients and 2.5% of matched non-endometriosis cohort patients (*p* = 0.888) had been diagnosed with breast cancer (Fig. 2).

No significant association between endometriosis and subsequent breast cancer (HR: 0.98; 95% CI 0.79–1.23) (Table 2) was observed in the regression analysis, nor were any significant associations observed in age-stratified analyses (Table 2).

**Table 2:** Association between endometriosis and subsequent breast cancer in women followed by office-based gynecologists in Germany (univariable Cox regression models)

Age group	Incidence (cases per 1000 patients years) in women with endometriosis	Incidence (cases per 1000 patients years) in women without endometriosis	HR (95% CI)	*p*-value
Total	2.4	2.4	0.98 (0.79–1.23)	0.888
Age ≤ 30	0.4	0.7	0.61 (0.22–1.71)	0.343
Age 31–40	1.9	1.6	1.16 (0.75–1.80)	0.497
Age 41–50	3.6	3.7	0.97 (0.70–1.34)	0.848
Age > 50	4.8	5.0	0.97 (0.58–1.62)	0.904

## Discussion

In this retrospective cohort study, we examined the association between endometriosis and the 10-year incidence of breast cancer in 15,242 women with endometriosis and an equal number of patients without endometriosis. To the best of our knowledge, this was the first cohort study from Germany investigating the potential association between endometriosis and subsequent breast cancer. The study found no significant association between endometriosis and subsequent breast cancer, both in the overall analysis and when considering individual age groups. Previous studies investigating the association between endometriosis and breast cancer have reported mixed results. In line with several publications, our study found that endometriosis was not associated with overall breast cancer risk [13, 24–26]. Nevertheless, other studies demonstrated a positive association between endometriosis and breast cancer [27–30]. In particular, Brinton et al. analyzed the risk of cancer after hospitalization for endometriosis using data from a nationwide Swedish inpatient register and found a significantly higher proportion of patients with breast cancer (SIR of 1.3, 95% CI 1.1–1.4), although it should be noted that the authors did not control for any confounders [24, 27]. In addition, Chuang and colleagues conducted a nested case–control study using data from the Taiwan National Health Insurance Research Database (NHIRD) and indicated that endometriosis (OR of 1.44, 95% CI 1.15–1.80) was associated with an increased breast cancer risk. Finally, the strongest association was demonstrated by Schairer et al., who examined a cohort of 15,844 women in the Uppsala health care region of Sweden who underwent gynecological surgery and revealed that endometriosis patients who had had a hysterectomy without ovarian ablation had a significantly higher breast cancer risk (SMR 3.2; 95% CI 1.2–8.0), whereas those who had had an oophorectomy without hysterectomy did not exhibit any significantly increased risk (SMR 1.7; 95% CI 0.7–4.1) [30, 31]. The inconsistency of the aforementioned study results may also be due to the occurrence of different biases as a result of confounding, missing data, criteria for participant selection, etc. In particular, the influence of factors such as infertility, which can arise from endometriosis and represents a risk factor for breast cancer, must be noted. As an example of missing data affecting study results, the use of oral contraceptives (including GnRH analogs), which are used in the treatment of endometriosis and also influence the risk of breast cancer, must be mentioned [1, 31].

There was also no association between endometriosis and breast cancer when individual age groups were considered. Given the median age at menopause among women from industrialized countries ranging between 50 and 52 years, it can also be assumed that menopausal status did not play any role in our study. Our results are consistent with previous studies, which also indicated that endometriosis is not associated with breast cancer regardless of menopausal status [13, 32]. However, one case–control study by Weiss et al. revealed an increased risk of breast cancer in premenopausal women with endometriosis (OR 1.99; 95% CI 1.0–4.0) [33].

We also found that the cumulative breast cancer incidence for patients with endometriosis was 2.4%, compared to 2.5% for those without endometriosis (Fig. 2). When comparing our national data from gynecological practices with cancer registry data from the Robert Koch Institute (RKI) in Germany, we found a slightly lower 10-year incidence rate for breast cancer in the latter. In particular, based on cancer registry data from 2018, breast cancer incidence in Germany in women aged 35 years was 1% (1 in 100) compared to 2% in women aged 45 years (1 in 46) [34]. Based on the mean age of 35.7 years in our study, it could be reasonable expect a slightly lower 10-year BC incidence. However, the incidence from office-based data may be higher than that arising from the cancer registry data due to good gynecological screening (e.g., breast sonography) by gynecologists [20]. Furthermore, it should be noted that the comparison of these data is limited by the lack of RKI cancer registry data for 2023.

Moreover, our data analysis revealed that patients visited their gynecologists an average of 3.2 times per year during the follow-up (Table 1). Even among patients treated by gynecologists, the time to endometriosis diagnosis is known to be relatively long, averaging a mean time of 4.4 years from symptom onset, and is accompanied by a high consultation frequency due to persistent pain [35]. Thus, consultation frequency needs to be considered as confounding when examining the association between endometriosis and BC in order to determine if an association between endometriosis and breast cancer incidence is a consequence of bias driven by better access of endometriosis patients to gynecological care. Analyzing the studies included in the meta-analysis by Kvaskoff et al. it becomes apparent that none of the studies included adjustments for consultation frequency [9]. Adjusted for this factor, our study can thus provide further evidence to clarify whether endometriosis patients have a higher breast cancer risk.

In summary, endometriosis was not found to be associated with a higher risk of overall breast cancer. Although the preliminary results are reassuring, gynecologists should continue to perform regular breast examinations on their endometriosis patients.

### Strengths and limitations

The major strengths of this retrospective study are the large sample size, the duration of follow-up (10 years), the medically confirmed diagnosis of endometriosis, the accurate adjustment for a plethora of factors (e.g., based on average annual consultation frequency during the follow-up period and an abundance of patients diagnosed with benign breast diseases), and the use of continuously updated data collected in 315 office-based gynecological practices in Germany. Nonetheless, the study findings should also be interpreted in the light of several limitations. Given that data generation is based on ICD codification, effects of erroneous coding, incorrect diagnoses, or possible effects due to modest changes in ICD categorization during the observational period cannot be dismissed. Second, diagnoses availability of further information on endometriosis (e.g., severity, subtype, symptoms) and breast cancer (e.g., TNM-classification, histological, and molecular subtype) would have allowed for more detailed analyses. Third, there was a lack of data on lifestyle factors (e.g., smoking status and consumption of alcohol) and anamnestic factors (e.g., family history of breast cancer, menopausal status, parity, hormone replacement, contraceptive use), although these factors may have impacted the cumulative incidence of breast cancer. Fourth, endometriosis and breast cancer have been diagnosed in specialized gynecological practices in Germany and the results of this study may not be extrapolated to other settings. Fifth, there is also a lack of hospital data and information on mortality.

## Data Availability

Anonymized raw data are available on reasonable request.
